# Hepatitis B virus genotype and basal core promoter/precore mutations are associated with hepatitis B-related acute-on-chronic liver failure without pre-existing liver cirrhosis

**DOI:** 10.1111/j.1365-2893.2009.01254.x

**Published:** 2010-12

**Authors:** X Ren, Z Xu, Y Liu, X Li, S Bai, N Ding, Y Zhong, L Wang, P Mao, F Zoulim, D Xu

**Affiliations:** 1 Viral Hepatitis Research Laboratory, Institute of Infectious DiseasesBeijing 302 Hospital, Beijing, China; 2 INSERM, U871 and Department of Hepatology and Gastroenterology, Hospices Civils de LyonHôtel Dieu, Lyon, France

**Keywords:** acute-on-chronic liver failure, basal core promoter, hepatitis B virus, mutation, precore

## Abstract

The study was undertaken to investigate the features and clinical implications of hepatitis B virus (HBV) genotypes, basal core promoter (BCP) and precore (PC) mutations in hepatitis B-related acute-on-chronic liver failure (HB-ACLF). Samples from 75 patients with HB-ACLF and without pre-existing liver cirrhosis and 328 age-matched patients with chronic hepatitis B (CHB) were analyzed. HBV genotype and BCP/PC mutations were determined by direct sequencing. Mutations at 8 sites of the BCP/PC region were compared between the two groups of patients. A significantly higher ratio of genotype B to C was found in patients with HB-ACLF than in patients with CHB (30.7–69.3%*vs*16.5–82.6%, *P* < 0.01). Single mutations including T1753V (C/A/G), A1762T, G1764A, G1896A and G1899A and triple mutations T1753V/A1762T/G1764A and A1762T/G1764A/C1766T (or T1768A) were more frequently detected in patients with HB-ACLF than in patients with CHB. Correspondingly, BCP/PC wild-type sequences were absent in patients with HB-ACLF in contrast to 27.1% in patients with CHB. The BCP/PC mutations were found to be associated with increased HBeAg negativity, higher alanine aminotransferase level and lower viral load. Patients with HB-ACLF infected with the PC mutant virus had a higher mortality. The findings suggest that patients with CHB infected with genotype B with BCP/PC mutations were more likely to develop HB-ACLF than those with genotype C with wild-type BCP/PC regions, and patients with HB-ACLF with the PC mutation had increased risk of a fatal outcome.

## Introduction

Hepatitis B virus (HBV) is responsible for chronic infection in about 350 million people worldwide and 93 million of them are in China [1,2]. Chronic HBV infection leads to a wide spectrum of clinical presentations ranging from an asymptomatic carrier state to chronic hepatitis B (CHB) with progression to liver cirrhosis (LC) and hepatocellular carcinoma (HCC) or to acute-on-chronic liver failure (ACLF). Recommendations for the definition of ACLF by the Asian Pacific Association for the Study of the Liver include acute hepatic insult characterized by jaundice and coagulopathy, complicated within 4 weeks by ascites and/or encephalopathy in a patient with previously diagnosed or undiagnosed chronic liver disease [3]. Similarly, in China, ACLF is defined as acute liver decompensation on the basis of chronic liver disease with mandatory jaundice [serum total bilirubin (TBIL) >171.0 μmol/L (i.e. >10.0 mg/dL) or a rapid rise >17.1 μmol/L/day (i.e. >1.0 mg/dL/day)] and coagulopathy [plasma prothrombin activity (PTA) <40%] and recent development of complications [4]. Different from western countries where alcoholic cirrhosis constitutes 50–70% of all underlying liver diseases in China, hepatitis B-related ACLF (HB-ACLF) cases account for more than 80% of ACLF cases as a result of the high incidence of chronic HBV infection [4,5]. HB-ACLF is reported to have a high mortality rate (60–80%) in the absence of liver transplantation, causing 22 600 deaths annually [6].

The pathogenesis of HB-ACLF remains largely unclear. Both viral and host factors may play a role. HBV is classified into eight genotypes (A–H) that may vary in geographical distribution, viral characteristics and relationship to clinical outcomes. HBV mutations in the basal core promoter (BCP) and precore (PC) have attracted special attention, because the BCP mutations may enhance HBV replication *in vitro* and the PC mutation abrogates translation of HBeAg, which is considered a tolerogen buffering any immune attack on the infected hepatocytes [7–9]. Studies have been performed to clarify these virologic features in patients with acute liver failure (ALF) who developed fulminant hepatitis from acute HBV infection. A higher prevalence of the BCP double mutation A1762T/G1764A and the G1896A PC mutation have been reported in ALF than in acute hepatitis B patients [10–15]. In addition, single mutations including the T1753V (C/A/G), C1766T, T1768A, G1862T and G1899A in the BCP/PC region have been reported to be associated with increased HBV replication capacity and/or reduced HBeAg expression *in vitro*, and in some cases associated with ALF in the clinic [7,15–19]. The influence of HBV genotypes on ALF has also been reported. Ozasa *et al.* [14] reported that patients infected with HBV/Bj were more likely to develop ALF compared to patients with HBV/Ae. However, inconsistent findings exist, showing no obvious link between HBV BCP/PC mutations and ALF or fulminant hepatitis development [20–23]. In some of these studies, a small sample size was used and genotypes were not defined. Bias therefore may partly account for the discrepancies in the conclusions from individual investigations.

Until now, little is known about the association of HBV genotypes and BCP/PC mutations with HB-ACLF development. Liu *et al.* [24] reported that these virologic features seemed not to associate with the fulminant exacerbation of CHB using a small sample size. Considering that the BCP/PC mutation prevalence may increase in LC patients [25,26], and older age is often associated with longer disease duration as persistent HBV infection is established mainly during infancy in most Asian countries [27], we analyzed viral genotype/subgenotype and BCP/PC mutations in a large number of patients with HB-ACLF and without pre-existing LC and age-matched patients with CHB in this study.

## Patients and methods

### Patients

Sera from 559 patients with HB-ACLF who were admitted to the Beijing 302 Hospital from January 2005 to December 2008 were tested. The patients came from various areas of China, but mainly from the North. Viral genes were successfully amplified and sequenced from both the BCP/PC and S regions from the sera of 298 cases. As most of these patients had pre-existing liver cirrhosis and thus were excluded, only 75 patients with HB-ACLF and without pre-existing LC were finally enroled. Three hundred and twenty-eight age-matched patients with CHB were taken as controls. The diagnostic criteria were based on the 2000 Xi’an viral hepatitis management scheme issued by the Chinese Society of Infectious Diseases and Parasitology, and the Chinese Society of Hepatology from the Chinese Medical Association [28]. All patients were seroposite for HBsAg for at least 6 months before enrolment. Patients with HB-ACLF met the following criteria: recent development of jaundice [TBIL >171.0 μmol/L (i.e. >10.0 mg/dL) or rapidly rising levels of >17.1 μmol/L/day (i.e. >1.0 mg/dL/day)] and decreasing PTA (<40%), with development of complications such as hepatic encephalopathy (≥grade 2), or abrupt and obvious increase in ascites or spontaneous bacterial peritonitis or hepatorenal syndrome. Patients with CHB met the following criteria: a history of chronic hepatitis based on histopathological diagnosis and/or compatible laboratory data and ultrasonographic findings, with mild to moderate liver inflammatory manifestations. For all patients, there was no evidence for LC, HCC or other metastatic liver disease; no evidence for concomitant HCV, HDV or HIV infection or autoimmune liver disease. Sera from patients with HB-ACLF were sampled once the diagnosis was made. The study was approved by the ethics committee of the Beijing 302 Hospital.

### Serological markers and quantification of hepatitis B virus DNA

Serum alanine aminotransferase (ALT), TBIL, albumin and other biochemical parameters were measured by standard procedures. HBeAg/anti-HBe, HBsAg/anti-HBs and anti-HBc were detected by enzyme-linked immunosorbent (Kewei Diagnostic Ltd., Beijing, China) or chemiluminescent assays (Abbott Laboratories, Chicago, IL, USA). HBV DNA level was determined by a real-time PCR kit (Fuxing Clone Co., Shanghai, China) with a lower limit of detection of 500 copies/mL (about 100 IU/mL).

### Detection of the basal core promoter/precore mutations

Viral DNA was extracted from 140 μL of serum using a Viral DNAout kit (Tiandz Engineering, Beijing, China). HBV genome sequences were determined by direct sequencing after PCR amplification. Primers for amplification of the BCP and PC regions were as follows: 5′-GAC GTC CTT TGT YTA CGT CC-3′ (nt 1413–1432) and 5′-TCT GCG ACG CGG CGA TTG AG-3′(antisense, nt 2403–2422) for the first-round PCR; 5′-ACT TCG CTT CAC CTC TGC AC-3′(sense, nt 1583–1602) and 5′-ATC CAC ACT CCA AAA GAY ACC-3′(antisense, nt 2257–2277) for the second-round PCR. The first-round PCR consisted of equilibrating at 94 °C for 3 min; 10 cycles at 94 °C for 35 s, 59 °C for 35 s (decreasing by 2 °C every other cycle), 72 °C for 70 s; and 30 cycles at 94 °C for 35 s, 56 °C for 35 s, and 72 °C for 70 s. The second-round PCR entailed a denaturation step at 94 °C for 3 min and 35 cycles at 94 °C for 25 s, 56 °C for 25 s and 72 °C for 50 s. The PCR products were separated on 1% agarose gels and purified by gel recovery. Sequencing was performed using an ABI 3730xl DNA Analyzer (Applied Biosystems, Foster City, CA, USA). Analysis and assembly of sequencing data were performed with the Vector NTI Suite software package (Informax, Frederick, MD, USA).

### Typing of hepatitis B virus genotypes/subgenotypes

The genotyping was based on S-gene sequences encompassing the reverse-transcriptase domain of HBV, which was amplified by an in-house nested PCR assay as previously described [2]. The entire 1225-bp fragments (nt 54–1278) amplified were directly sequenced. HBV genotype and subgenotype were determined by molecular evolutionary analysis of the viral sequences using the MEGA 4 software. Phylogenetic trees were constructed using neighbour-joining (NJ) analysis with bootstrap test confirmation performed on 1000 resamplings. Standard reference sequences were acquired from the online Hepatitis Virus Database (http://www.ncbi.nlm.nih.gov/projects/genotyping/formpage.cgi) as previously reported [29].

### Statistical analysis

Values for results were expressed as means ± standard deviation or median. Differences in data between two groups were examined by chi-square test, Fisher’s exact test, Student’s *t*-test or nonparametric Mann–Whitney *U* test where appropriate. Statistical analysis was carried out in SPSS 16.0 software. A *P* value of <0.05 was considered statistically significant.

## Results

### Clinical background, hepatitis B virus genotype/subgenotype and basal core promoter/precore mutation profiles of the studied patients

Table 1 summarizes the clinical background and the virologic characteristics (HBV genotypes, BCP/PC mutations) of the 75 HB-ACLF and 328 patients with CHB. Genotype B (B1, 2.6%; B2, 92.2%; B3, 1.3%; B4, 1.3%; undetermined, 2.6%), C (C1, 1.6%; C2, 96.6%; C3, 1.2%; C4, 0.3%; undetermined, 0.3%) and D were detected in 19.1%, 80.1% and 0.8% of patients, respectively. A significantly higher ratio of genotype B to C was found in HB-ACLF than in patients with CHB (30.7–69.3%*vs* 16.5–82.6%, *P*<0.01). Alternatively, 29.9% (23/77) of the patients with genotype B virus infection had HB-ACLF compared to 16.1% (52/323) of patients with genotype C infection. A significantly higher prevalence of the T1753V, A1762T, G1764A, G1896A and G1899A mutations was detected in HB-ACLF than in patients with CHB. Wild-type BCP/PC sequences (at the 8 mutation sites analyzed) were found in none of the patients with HB-ACLF, but such sequences were present in 27.1% of patients with CHB.

**Table 1:** Clinical background, HBV genotype and BCP/PC mutation profiles of the patients studied

	Total (*n* = 403)	HB-ACLF (*n* = 75)	CHB (*n* = 328)	*P* value
Gender (M/F)	348/55	67/8	281/47	0.405
Age (years)	39 (18–75)	39 (19–72)	38 (18–75)	0.273
Total bilirubin (μmol/L)	15.3 (3.8–1053)	451.0 (191.1–1053)	12.8 (3.8–77)	<0.001
ALT (IU/L)	50 (7–3774)	569 (57–3774)	41(7–665)	<0.001
HBV DNA (logcps/mL)	5.26 ± 1.6	5.3 ± 1.7	5.2 ± 1.6	0.702
Prothrombin activity (%)	75.0 (5.0–238.9)	22.7 (5–42)	87.4 (43.6–238.9)	<0.001
Albumin	41.5 ± 7.65	29.9 ± 3.45	44.2 ± 5.52	<0.001
Albumin/globulin ratio	1.58 ± 0.46	1.23 ± 0.64	1.66 ± 0.36	<0.001
HBeAg+	223 (55.8%)	29 (38.7%)	194 (59.1%)	0.001
Anti-HBe+	122 (30.3%)	27 (36.0%)	95 (29.0%)	0.231
Genotype B	77 (19.1%)	23 (30.7%)	54 (16.5%)	0.009
Genotype C	323 (80.1%)	52 (69.3%)	271 (82.6%)	0.009
Genotype D	3 (0.8%)	0	3 (0.9%)	–
Wild type	89 (22.1%)	0	89 (27.1%)	<0.001
T1753V (C/A/G)	72 (17.9%)	21 (28%)	51 (15.5%)	0.012
T1754G	11 (2.7%)	3 (4.0%)	8 (2.4%)	0.193
A1762T	230 (57.1%)	58 (77.3%)	172 (52.4%)	<0.001
G1764A	239 (59.3%)	62 (82.7%)	177 (54.0%)	<0.001
C1766T	21 (5.2%)	5 (6.7%)	16 (4.9%)	0.529
T1768A	10 (2.5%)	2 (2.7%)	8 (2.4%)	0.584
G1896A	138 (34.2%)	34 (45.3%)	105 (32.0%)	0.038
G1899A	34 (8.4%)	12 (16.0%)	22 (6.7%)	0.013

HB-ACLF, hepatitis B-related acute-on-chronic liver failure; ALT, alanine aminotransferase; BCP, basal core promoter; CHB, chronic hepatitis B; HBV, hepatitis B virus; PC, precore.

### Individual profiles of basal core promoter/precore mutations in patients infected with genotypes B and C

Genotype B had a significantly lower prevalence of BCP mutations T1753V, A1762T and G1764A but significantly higher prevalence of the G1896A PC mutation in comparison with genotype C. The C1766T and T1768A mutations were not found in the genotype B samples studied. The average number of substitutions per sample at the eight analyzed sites was significantly higher in genotypes C than B samples (Table 2).

**Table 2:** Comparison of the BCP/PC mutation occurrence between genotypes B and C

	Genotype B (*n* = 77)	Genotype C (*n* = 323)	*P* value
T1753V	4 (5%)	68 (21.1%)	<0.001
T1754G	5 (6%)	6 (2%)	0.025
A1762T	29 (37.7%)	201 (62.2%)	<0.001
G1764A	29 (37.7%)	210 (65.0%)	<0.001
C1766T	0	21 (6.5%)	–
T1768A	0	10 (3.1%)	–
G1896A	36 (46.7%)	102 (31.6%)	0.012
G1899A	7 (9%)	27 (8.4%)	0.836
Substitution/sample*	1.48	2.06	0.001

BCP, basal core promoter; PC, precore.

* Represents average number of substitutions at the eight analyzed sites from the BCP/PC regions.

Table 3 summarizes the profiles of the BCP/PC mutations in patients infected with genotypes B or C separately. In genotype B infection, a statistical difference in the mutation occurrence between patients with HB-ACLF and patients with CHB was only observed at the A1762T and G1764A sites. By contrast, in genotype C infection, a significantly higher mutation prevalence was observed at the 1753, 1762, 1764, 1896 and 1899 sites in patients with HB-ACLF in comparison with patients with CHB. Nevertheless, the average number of substitutions per sample was significantly higher in HB-ACLF than in patients with CHB for both genotypes B and C infections.

**Table 3:** Individual profiles of BCP/PC mutations in patients infected with genotypes B and C

	HB-ACLF (*n* = 75)	CHB (*n* = 328)	*P* value
Genotype B (*n* = 77)			
Patient number	23	54	
Wild type	0	21	<0.001
T1753V	2 (8.7%)	2 (3.7%)	0.366
T1754G	2 (8.7%)	3 (5.6%)	0.714
A1762T	14 (60.9%)	15 (27.8%)	0.006
G1764A	14 (60.9%)	15 (27.8%)	0.006
C1766T	0	0	–
T1768A	0	0	–
G1896A	13 (56.5%)	23 (42.6%)	0.262
G1899A	3 (13.0%)	4 (7.4%)	0.431
Substitution/sample*	2.17	1.19	<0.001
Genotype C (*n* = 323)			
Patient number	52	271	
Wild type	0	68	<0.001
T1753C/A/G	19 (36.5%)	49 (18.1%)	<0.001
T1754C/G	1 (1.9%)	5 (1.8%)	0.654
A1762T	44 (84.6%)	157 (57.9%)	<0.001
G1764A	48 (92.3%)	162 (59.8%)	<0.001
C1766T	5 (9.6%)	16 (5.9%)	0.320
T1768A	2 (3.8%)	8 (3.0%)	0.733
G1896A	20 (38.5%)	82 (30.3%)	0.049
G1899A	9 (17.3%)	18 (6.6%)	0.002
Substitution/sample*	2.92	1.88	<0.001

HB-ACLF, hepatitis B-related acute-on-chronic liver failure; BCP, basal core promoter; CHB, chronic hepatitis B; PC, precore.

* Represents the average number of substitutions at the eight analyzed sites from the BCP/PC regions.

### Hepatitis B virus basal core promoter/precore mutational patterns and disease status

To simplify data analysis, we defined the A1762T/G1764A and G1896A as the basic BCP and basic PC mutation, respectively, as these were well-known hotspot mutations. Accordingly, there were four basic patterns, i.e. no mutations (BCP−/PC−), BCP mutation only (BCP+/PC−), PC mutation only (BCP−/PC+) and both mutations (BCP+/PC+). In addition, the latter two patterns were also analyzed together as BCP±/PC+. A significantly lower frequency of the BCP−/PC− pattern and a higher frequency of the BCP+/PC− and BCP±/PC+ patterns were found in patients with HB-ACLF in comparison with patients with CHB. In addition, two interesting triple BCP mutations [T1753V/A1762T/G1764A and A1762T/G1764A/C1766T (or T1768A)] were more frequently detected in HB-ACLF than in patients with CHB (Table 4).

**Table 4:** HBV BCP/PC patterns distributed in the two groups of patients

	HB-ACLF	CHB	*P* value
Basic BCP/PC mutations*	*n* = 75	*n* = 328	
−/−	5 (6.7%)	119 (36.2%)	<0.001
+/−	37 (49.3%)	104 (31.7%)	0.003
−/+	12 (16.0%)	41 (12.5%)	0.262
+/+	21 (28.0%)	64 (19.5%)	0.073
±/+	33 (44.0%)	105 (32.0%)	0.034
Triple BCP mutations†	*n* = 52	*n* = 271	
T1753V/A1762T/G1764A	19 (36.5%)	48 (17.7%)	0.002
A1762T/G1764A/C1766T (or T1768A)	6 (11.5%)	11 (4.1%)	0.027

HB-ACLF, hepatitis B-related acute-on-chronic liver failure; BCP, basal core promoter; CHB, chronic hepatitis B; HBV, hepatitis B virus; PC, precore.

* Positivity (+) or negativity (−) of basic BCP and PC mutations was based on the presence or absence of A1762T/G1764A and G1896A, respectively.

† Only genotype C HBV-infected patients were analyzed. A1762T/G1764A/C1766T (or T1768A) may be concomitant with T1753V.

### Hepatitis B virus basal core promoter/precore mutational patterns in relation to alanine aminotransferase and hepatitis B virus DNA levels, and HBeAg negativity

**Fig. 1:**
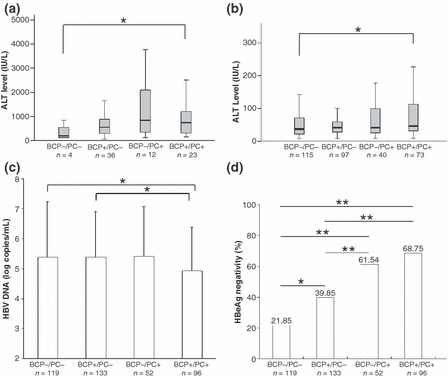
HBV BCP/PC mutational patterns in relation to alanine aminotransferase (ALT) and HBV DNA levels, and HBeAg negativity. Four patterns (BCP−/PC−, BCP+/PC−, BCP−/PC+ and BCP+/PC+) were determined based on positivity (+) or negativity (−) for the BCP double mutation A1762T/G1764A and the G1896A PC mutation. Association of the BCP/PC patterns with (a) serum ALT level in patients with hepatitis B-related acute-on-chronic liver failure (HB-ACLF); (b) serum ALT level in patients with chronic hepatitis B (CHB); (c) serum HBV DNA level in all patients; (d) HBeAg negative rate in all patients. Data are expressed as box plots, in which the horizontal lines illustrate the 25th, 50th and 75th percentiles for ALT level, and mean ± SD for HBV DNA level and HBeAg negative rate, respectively. *P<0.05, **P<0.01. BCP, basal core promoter; HBV, hepatitis B virus; PC, precore.

As ALT level was significantly different between HB-ACLF and CHB groups (Table 1), its association with the basic BCP/PC patterns was analyzed separately for the two groups of patients. The results showed that ALT was significantly higher in both patients with HB-ACLF and with CHB infected with the BCP+/PC+ virus than in those with the BCP−/PC− one (Figs 1a,b). With respect to HBV DNA, as there was no significant difference between HB-ACLF and CHB groups (Table 1), its association with the basic BCP/PC patterns was analyzed together for the two groups of patients. The results showed that patients with BCP+/PC+ virus had lower HBV DNA level than those with BCP−/PC− or BCP+/PC− viruses (Fig. 1c). The HBeAg negative rate increased in a stepwise manner in patients with BCP−/PC− (21.8%), BCP+/PC− (39.8%), BCP−/PC+ (61.5%) and BCP+/PC+ (68.8%) viruses (Fig. 1d).

### The clinical and virologic characteristics in relation to mortality in patients with hepatitis B-related acute-on-chronic liver failure

Of the 75 patients with HB-ACLF, 42 had a fatal outcome and 33 survived more than 6 months after onset of liver failure. Positive BCP/PC mutations, higher TBIL and lower PTA were detected as risk factors, while a high viral load (≥10^5^ copies/mL) was only marginally significant as a risk factor for mortality. In addition, there was a trend that infection with genotype B was associated with fatal outcome. Older age (≥40 years old) and HBeAg negativity were not associated with survival rate (Table 5).

**Table 5:** The clinical and viral characteristics in relation to mortality in patients with hepatitis B-related acute-on-chronic liver failure

Factors	Survival	Nonsurvival	*P* value	Odds ratio
Basic BCP/PC mutations				
−/−	2	3	–	
+/−	21	16	0.028	0.35 (0.14–0.90)
±/+	10	23	0.026	3.02 (1.1–8.10)
HBeAg				
+	15	23	0.424	1.45 (0.58–3.63)
−	18	19		1
Total bilirubin				
<450 μmol/L	21	15	0.016	1
≥450 μmol/L	12	27		3.15 (1.22–8.14)
Prothrombin activity (%)				
<23	11	27	0.007	3.60 (1.38–9.41)
≥23	22	15		1
HBV DNA				
<10^5^ copies/mL	11	6	0.050	1
≥10^5^ copies/mL	22	36		3.00 (0.97–9.26)
Genotype				
B	7	16	0.092	2.29 (0.81–6.48)
C	26	26		1
Age				
<40 years	19	18	0.151	1
≥40 years	14	24		1.81 (0.72–4.55)

BCP, basal core promoter; HBV, hepatitis B virus; PC, precore.

## Discussion

The outcome of HBV infection depends on the interplay between the virus, the hepatocytes and the host’s immune response. When the balance is disrupted by the emergence of particular HBV mutants with altered phenotype, the changed virus–cell relationship might cause hepatocyte necrosis and lead to the development of fulminant or severe hepatitis [30]. Thus, the investigation of the virologic features that may help explain the pathogenesis and give potential indicators of HB-ACLF development may prove useful. HB-ACLF is different from fulminant hepatitis on the basis of acute HBV infection. It is also different from acute exacerbation of CHB, which is defined as clinical symptoms along with an abrupt rise in serum ALT to a certain level, e.g. above 200 IU/L [31] or 500 IU/L [32], and only a small proportion of such patients develops fulminant hepatic failure that meets the criteria of HB-ACLF. Patients with HB-ACLF are relatively rare compared to the large population of patients with CHB in China, and usable samples for the analysis were even fewer because the sensitivity of the BCP/PC amplification was obviously reduced as shown here. The Beijing 302 Hospital is one of the largest hospitals for infectious and liver diseases in China and is well known for the management of hepatitis B. Patients from various areas of China come to the hospital-seeking treatment, and this allowed us to collect a larger sample size representing patients with HB-ACLF.

Our results showed that genotype B infection was more frequently observed in HB-ACLF than in patients with CHB, suggesting that patients with CHB infected with genotype B have a higher risk of developing liver failure than those infected with genotype C. A significantly higher occurrence of BCP mutations at the 1753, 1762 and 1764 sites, and PC mutations at the 1896 and 1899 sites were found in patients with HB-ACLF than in patients with CHB (Table 1), suggesting that accumulation of BCP/PC mutations could be a potential indicator and probably a contributor to HB-ACLF development. Further analysis indicated that the BCP mutations T1753V, A1762T and G1764A were more frequently detected in genotype C than in genotype B isolates, whereas the G1896A PC mutation was more frequently detected in genotype B than in genotype C ones (Table 2), confirming previous reports [33,34]. In patients infected with genotype C, a comparable increase in the frequency of BCP/PC mutations was obtained as for the total patient sample; whereas in patients infected with genotype B, a significant difference was only observed at the 1762 and 1764 sites in the BCP region. This may be partly because of a lower number of genotype B infected patients enroled and partly because of particular genetic features of the viral genotypes. Nevertheless, the average number of substitutions in the BCP/PC region were significantly increased in the HB-ACLF compared to CHB group regardless of virus genotype, suggesting that accumulation of the BCP/PC mutations increased the risk of HB-ACLF occurrence in general.

The A1762T/G1764A and G1896A well-recognized BCP/PC mutations were defined as basic BCP and PC mutations in our study. Patients with HB-ACLF had a significantly higher prevalence of basic BCP mutations alone or in combination with the basic PC mutation in comparison with patients with CHB. Correspondingly, virus without basic BCP and PC mutations was much less frequently detected in patients with HB-ACLF than in patients with CHB (Table 4). It has been reported that combined A1762T/G1764A mutations with T1753V, C1766T and/or T1768A may enhance viral replication *in vitro* and are associated with ALF and advanced liver disease [35–37]. Therefore, we analyzed two triple BCP mutational patterns T1753V/A1762T/G1764A and A1762T/G1764A/C1766T (or T1768A) in patients infected with HBV genotype C, as T1753V was detected infrequently in genotype B and not at all in C1766T and T1768A. As a result, both BCP triple mutational patterns were present significantly more so in HB-ACLF than in patients with CHB, irrespective of whether the G1896A PC mutation was there or not (Table 4). These data may suggest that emergence of the T1753V, C1766T and T1768A mutations was likely an additional driving factor for the development of HB-ACLF.

Elevated ALT level was found to be associated with the emergence of the basic BCP/PC mutations, both in ACLF and patients with CHB (Figs 1a,b). By contrast, HBV DNA level was decreased with the emergence of the basic BCP/PC mutations (Fig. 1c). It might be speculated that the BCP/PC mutant viruses with enhanced viral replication and reduced/abrogated HBeAg expression were more likely to activate immune responses, leading to ALT elevation and viral load decline in patients with CHB. In cases where the virus-host balance is interrupted, a robust and out-of-control inflammatory response is triggered that may lead to extensive hepatic injury. However, the alternation of ALT and HBV DNA levels may be influenced by the use of antiviral treatment and a blood test at a single time-point may give a biased evaluation of disease activity for some patients with CHB with fluctuating ALT and HBV DNA levels, although these influences were relatively proportional in the larger sample size of the current study. As expected, the HBeAg negative rate increased in a stepwise manner along with the emergence of the basic BCP mutation, PC mutation or both (Fig. 1d). As mutant viruses frequently coexist with wild-type viruses, subpopulations comprising <20% of the total HBV population may be missed by direct sequencing techniques [38]. Thus, the G1896A mutants can sometimes be detected in HBeAg positive patients if they are present at higher levels.

With respect to mortality of patients with HB-ACLF in our study, infection with the BCP/PC mutants was associated with nonsurvival in addition to biochemical parameters such as PTA and TBIL levels. In addition, there was a tendency towards a fatal outcome in patients with HB-ACLF who had high HBV DNA levels (≥10^5^ copies/mL) and genotype B infection. Intriguingly, emergence of the BCP mutation alone seemed to have a protective effect on the occurrence of HB-ACLF. Older age (≥40 years old) was not an independent factor that was associated with poor outcome. This is not consistent with some previous results as in the King’s College and Clichy criteria [39,40]. This could be explained by differences in aetiology and clinical status of the patients enroled in different investigations. In our study, exclusion of ACLF cases with pre-existing LC minimized any influences from complex aetiology.

In summary, HBV genotype and BCP/PC mutations were associated with development of HB-ACLF. Patients with CHB infected with genotype B and BCP/PC mutant virus were more prone to develop HB-ACLF than those with genotype C and BCP/PC wild-type virus. Patients with HB-ACLF and with PC mutant virus had an increased risk of having a fatal outcome. Some of these findings are similar as those found in ALF and acute hepatitis B patients, suggesting that HBV genotype and BCP/PC mutations might comparably be involved in the pathogenesis of fulminant hepatitis on the basis of both acute and chronic HBV infection. The study represents the first step towards understanding of the virologic factors that may contribute to the development of HB-ACLF.

## Abbreviations:

HBV: hepatitis B virus
BCP: basal core promoter
PC: precore
HB-ACLF: hepatitis B-related acute-on-chronic liver failure
ALF: acute liver failure
CHB: chronic hepatitis B
LC: liver cirrhosis
HCC: hepatocellular carcinoma
ALT: alanine aminotransferase
TBIL: total bilirubin
PTA: prothrombin activity
PCR: polymerase chain reaction

## References

[1] European association for the study of the liver (2009). EASL clinical practice guidelines: management of chronic hepatitis B. J Hepatol.

[2] Liu Y, Wang CM, Cheng J (2009). Hepatitis B virus in tenofovir-naive Chinese patients with chronic hepatitis B contains no mutation of rtA194T conferring a reduced tenofovir susceptibility. Chin Med J.

[3] Sarin SK, Kumar A, Almeida JA (2009). Acute-on-chronic liver failure; consensus recommendations of the Asian Pacific Association for the Study of the Liver (APASL). Hepatol Int.

[4] Liver Failure and Artificial Liver Group, Chinese Society of Infectious Diseases, Chinese Medical Association; Severe Liver Diseases and Artificial Liver Group, Chinese Society of Hepatology (2006). Diagnostic and treatment guidelines for liver failure. Chin J Hepatol (Chinese).

[5] Zou Z, Li B, Xu D (2008). Imbalanced intrahepatic cytokine expression of interferon-γ, tumor necrosis factor-α, and interleukin-10 in patients with acute-on-chronic liver failure associated with hepatitis B virus infection. J Clin Gastroenterol.

[6] Liu Q, Liu Z, Wang T, Wang Q, Shi X, Dao W (2007). Characteristics of acute and sub-acute liver failure in China: nomination, classification and interval. J Gastroenterol Hepatol.

[7] Baumert TF, Rogers SA, Hasegawa K, Liang TJ (1996). Two core promoter mutation identified in a hepatitis B virus strain associated with fulminant hepatitis result in enhanced viral replication. J Clin Invest.

[8] Tong S, Kim KH, Chante C, Wands J, Li J (2005). Hepatitis B virus e antigen variants. Int J Med Sci.

[9] Kay A, Zoulim F (2007). Hepatitis B virus genetic variability and evolution. Virus Res.

[10] Kosaka Y, Takase K, Kojima M (1991). Fulminant hepatitis B: induction by hepatitis B virus mutants defective in the precore region and incapable of encoding e antigen. Gastroenterology.

[11] Sato S, Suzuki K, Akahane Y (1995). Hepatitis B virus strains with mutations in the core promoter in patients with fulminant hepatitis. Ann Intern Med.

[12] Inoue K, Yoshiba M, Sekiyama K, Okamoto H, Mayumi M (1998). Clinical and molecular virological difference between fulminant hepatic failures following acute and chronic infection with hepatitis B virus. J Med Virol.

[13] Friedt M, Gerner P, Lausch E, Trübel H, Zabel B, Wirth S (1999). Mutations in the basic core promotor and the precore region of hepatitis B virus and their selection in children with fulminant and chronic hepatitis B. Hepatology.

[14] Ozasa A, Tanaka Y, Orito E (2006). Influence of genotypes and precore mutations on fulminant or chronic outcome of acute hepatitis B virus infection. Hepatology.

[15] Liang TJ, Hasegawa K, Rimon N, Wands JR, Ben-Porath E (1991). A hepatitis B virus mutant associated with an epidemic of fulminant hepatitis. N Engl J Med.

[16] Parekh S, Zoulim F, Ahn SH (2003). Genome replication, virion secretion, and e antigen expression of naturally occurring hepatitis B virus core promoter mutants. J Virol.

[17] Hou J, Lin Y, Waters J (2002). Detection and significance of a G1862T variant of hepatitis B virus in Chinese patients with fulminant hepatitis. J Gen Virol.

[18] Sainokami S, Abe K, Sato A (2007). Initial load of hepatitis B virus (HBV), its changing profile, and precore/core promoter mutations correlate with the severity and outcome of acute HBV infection. J Gastroenterol.

[19] Wai CT, Fontana RJ, Polson J, Hussain M, Shakil AO, Han SH (2005). Clinical outcome and virological characteristics of hepatitis B-related acute liver failure in the United States. J Viral Hepat.

[20] Sterneck M, Günther S, Santantonio T (1996). Hepatitis B virus genomes of patients with fulminant hepatitis do not share a specific mutation. Hepatology.

[21] Yuasa R, Takahashi K, Dien BV (2000). Properties of hepatitis B virus genome recovered from Vietnamese patients with fulminant hepatitis in comparison with those of acute hepatitis. J Med Virol.

[22] Chun YK, Kim JY, Woo HJ (2005). No significant correlation exists between core promoter mutations, viral replication and liver damage in chronic hepatitis B infection. Hepatology.

[23] Gandhe SS, Chadha MS, Walimbe AM, Arankalle VA (2003). Hepatitia B virus: prevalence of precore/core promoter mutants in different clinical categories of Indian patients. J Viral Hepat.

[24] Liu CJ, Kao JH, Lai MY, Chen PJ, Chen DS (2004). Precore/core promoter mutations and genotypes of hepatitis B virus in chronic hepatitis B patients with fulminant or subfulminant hepatitis. J Med Virol.

[25] Günther S, Piwon N, Iwanska A, Schilling R, Meisel H, Will H (1996). Type, prevalence, and significance of core promoter/enhancer II mutations in hepatitis B viruses from immunosuppressed patients with severe liver disease. J Virol.

[26] Preikschat P, Günther S, Reinhold S (2002). Complex HBV populations with mutations in core promoter, C gene, and pre-S region are associated with development of cirrhosis in long-term renal transplant recipients. Hepatology.

[27] Sugauchi F, Orito E, Ohno T (2006). Spatial and chronological differences in hepatitis B virus genotypes from patients with acute hepatitis B in Japan. Hepatol Res.

[28] Chinese Society of Infectious Diseases and Parasitology, and the Chinese Society of Hepatology, of the Chinese Medical Association (2000). Management scheme of diagnostic and therapy criteria of viral hepatitis. Zhonghua Gan Zang Bing Za Zhi (Chin J Hepatol).

[29] Fang ZL, Sabin CA, Dong BQ (2009). The association of HBV core promoter double mutations (A1762T and G1764A) with viral load differs between HBeAg positive and anti-HBe positive individuals: a longitudinal analysis. J Hepatol.

[30] Bartholomeusz A, Locarnini S (2001). Hepatitis B virus mutants and fulminant hepatitis B: fitness plus phenotype. Hepatology.

[31] Tsai WL, Lo GH, Hsu PI (2008). Role of genotype and precore/basal core promoter mutations of hepatitis B virus in patients with chronic hepatitis with acute exacerbation. Scand J Gastroenterol.

[32] Kusumoto K, Yatsuhashi H, Nakao R, Hamada R, Fukuda M, Tamada Y (2008). Detection of HBV core promoter and precore mutations helps distinguish flares of chronic hepatitis from acute hepatitis B. J Gastroenterol Hepatol.

[33] Yuen MF, Sablon E, Tanaka Y (2004). Epidemiological study of hepatitis B virus genotypes, core promoter and precore mutations of chronic hepatitis B infection in Hong Kong. J Hepatol.

[34] Kramvis A, Kew MC (2005). Relationship of genotypes of hepatitis B virus to mutations, disease progression and response to antiviral therapy. J Viral Hepat.

[35] Chauhan R, Kazim SN, Bhattacharjee J, Sakhuja P, Sarin SK (2006). Basal core promoter, precore region mutations of HBV and their association with e antigen, genotype, and severity of liver disease in patients with chronic hepatitis B in India. J Med Virol.

[36] Jammeh S, Tavner F, Watson R, Thomas HC, Karayiannis P (2008). Effect of basal core promoter and pre-core mutations on hepatitis B virus replication. J Gen Virol.

[37] Guo X, Jin Y, Qian G, Tu H (2008). Sequential accumulation of the mutations in core promoter of hepatitis B virus is associated with the development of hepatocellular carcinoma in Qidong, China. J Hepatol.

[38] Kalinina T, Riu A, Fischer L, Santantonio T, Will H, Sterneck M (2003). Selection of a secretion-incompetent mutant in the serum of a patient with severe hepatitis B. Gastroenterology.

[39] O’Grady JG, Alexander GJ, Hayllar KM, Williams R (1989). Early indicators of prognosis in fulminant hepatic failure. Gastroenterology.

[40] Bernuau J, Goudeau A, Poynard T (1986). Multivariate analysis of prognostic factors in fulminant hepatitis B. Hepatology.

